# *SP110* and *PMP22* polymorphisms are associated with tuberculosis risk in a Chinese-Tibetan population

**DOI:** 10.18632/oncotarget.11847

**Published:** 2016-09-06

**Authors:** Guoxia Ren, Jiangtao You, Xianfeng Gong, Xiucheng Zhang, Lin Zhao, Xianglan Wei, Tianbo Jin, Mingwei Chen

**Affiliations:** ^1^ Department of Respiratory and Critical Care Medicine, The First Affiliated Hospital of School of Medicine of Xi'an Jiaotong University, Xi'an 710061, People's Republic of China; ^2^ Department of Intergrated Traditional Chinese and Western Medicine, Xi'an Chest Hospital, Xi'an 710061, People's Republic of China; ^3^ Department of Thoracic Surgery, First Affiliated Hospital of Xi'an Jiaotong University, Xi'an 710061, People's Republic of China; ^4^ School of Life Sciences, Northwest University, Xi'an 710069, People's Republic of China; ^5^ Key Laboratory of High Altitude Environment and Genes Related to Diseases of Tibet Autonomous Region, School of Medicine, Tibet University for Nationalities, Xianyang 712082, People's Republic of China; ^6^ Xi'an Tiangen Precision Medical Institute, Xi'an 710075, People's Republic of China

**Keywords:** SP110, PMP22, single nucleotide polymorphism (SNP), tuberculosis (TB), Tibet

## Abstract

Susceptibility to tuberculosis (TB) is partially dependent on host genetic variability. *SP110* and *PMP22* are candidate genes identified in this study as associated with human susceptibility to TB. Here we performed an association analysis in a case-control study of a Tibetan population (217 cases and 383 controls). Using bioinformatics methods, we identified two SNPs in *SP110* that may decrease susceptibility to TB (rs4327230, *p*<0.001, OR: 0.37, 95%CI: 0.25-0.55; rs2114591, *p*<0.001, OR: 0.59, 95%CI: 0.45-0.78), whereas one SNP in *PMP22* appeared to increase TB risk (rs13422, *p*=0.003, OR: 1.45, 95%CI: 1.14-1.84). SNPs rs4327230 and rs2114591 remained significant after Bonferroni correction (*p*<0.00178). We found that the “GC” haplotype in *SP110* was protective against TB, with a 64% reduction in disease risk. “CA” and “CG” in *PMP22* were also associated with a protective effect. Our study indicates there is an association between specific gene polymorphisms and TB risk in a Tibetan population, and may help to identify those TB-affected individuals most susceptible to disease.

## INTRODUCTION

Tuberculosis (TB) is a highly infectious respiratory disease that remains one of the world's deadliest communicable diseases. According to the World Health Organization (WHO), an estimated 9 million people developed TB and 1.5 million died of the disease in 2013. Although one third of the world's population is infected with the bacillus *Mycobacterium tuberculosis*, the causative agent of TB, only about 10% will eventually develop clinical disease [1]. Whether or not an individual ultimately develops active disease depends upon both the host immune response and environmental aspects [2]. Genetic factors play an important role in immune system function and are thought to be the key factor in determining an individual's susceptibility to TB [2-5].

The *SP110* gene on chromosome 2 encodes a leukocyte-specific nuclear body component that can function as an activator of gene transcription and may serve as a nuclear hormone receptor coactivator. *SP110* is a promising candidate gene in the regulation of individual susceptibility to *M. tuberculosis*. First reported by Pan H, *et al.* [6], *SP110* is the closest human homolog of the mouse gene *Ipr1* (Intracellular Pathogen Resistance 1), which protects against tuberculosis by encoding Speckled proteins (SP) that regulate cell activation, division and apoptosis [7]. Previous studies associated *SP110* polymorphisms and susceptibility to TB in north Indian [8], Vietnamese [9] and Chinese Han [10] populations, although some groups reported negative results [11, 12]. In addition, the *PMP22* (peripheral myelin protein 22) gene on chromosome 17, previously reported to be associated with Charcot Marie Tooth (CMT) disease or hereditary neuropathy with pressure palsies (HNPP), may also play an important role in susceptibility to TB [13-15]. However, neither *SP110* nor *PMP22* has yet been investigated with respect to TB in the Tibetan population.

To investigate the association between *SP110* and *PMP22* and TB risk in Tibetans, we performed an association analysis in a case-control study. Our findings will help to improve patient-specific TB treatment and diagnosis options in the Tibetan population.

## RESULTS

We recruited 217 cases (118 males and 99 females; average age at diagnosis: 49.9) and 383 controls (153 males and 230 females; average age: 20.3) for this study (Table 1). Multivariate analyses were adjusted for age and sex.

**Table 1:** Patient demographics

Variables	Case (N=217)	Control (N=383)	Total	*p*-value
Sex, No.(%)				< 0.05^a^
Male	118 (43.3)	153 (60)	287 (52.5)	
Female	99 (56.7)	230 (40)	260 (47.5)	
Mean age ±SD	33.2 ±2.75	49.9 ±5.14		< 0.05^b^

a *P* values was calculated from Pearson's chi-square tests.

b *P* values was calculated by Welch's t tests.

To design the multiplexed SNPs MassEXTEND assay, we used Sequenom MassARRAY Assay Design 3.0 Software (PCR primers are shown in Table 2). A total of seven SNPs (four in *SP110* and three in *PMP22*) were identified in cases and controls. All SNP call rates exceeded 98.2%, which was considered high enough to perform association analyses. SNPs in controls were all in Hardy-Weinberg equilibrium (HWE) (Table 3). We found three SNPs in *SP110* (rs6436917, *p*=0.022, OR: 1.32, 95%CI: 1.04–1.67; rs4327230, *p*<0.001, OR: 0.37, 95%CI: 0.25–0.55; rs2114591, *p*<0.001, OR: 0.59, 95%CI: 0.45–0.78) and one in *PMP22* (rs13422, *p*=0.003, OR: 1.45, 95%CI: 1.14–1.84) that exhibited differential allele frequency distributions in cases vs. controls. Rs4327230 and rs2114591 in *SP110* remained significant after Bonferroni correction (*p*<0.00178).

**Table 2:** PCR primers used in this study

SNP_ID	1st-PCR primer	2nd-PCR primer	UEP_SEQ
rs6436917	ACGTTGGATGGCCAATTGTAAGTGCCAAAG	ACGTTGGATGATGGCCCTAAAATGTTCCAC	ggAATGTTCCACAGTGGGC
rs4327230	ACGTTGGATGACAATAGCAAAGACATGGGC	ACGTTGGATGTATGGCTGCATAGTATTCCG	ACATATTCTTTATCCAGTCTATAATT
rs2114591	ACGTTGGATGGAGACGTACAGAAAAGAGGG	ACGTTGGATGCTTCCAACAACCACTGTCAC	aacaACTGTCACATCAACAGATCC
rs7580912	ACGTTGGATGACACCCTCTCTCCTAACTAC	ACGTTGGATGGTCTCAAACCACAAACCACC	aaaaaCACAAACCACCAGGGCTTCC
rs13422	ACGTTGGATGTTGGGATTTTGGGCTAGCTC	ACGTTGGATGTCTGAGCGTACATAGGGAAG	ggagcGGGAAGGGAGGAAGGGAAA
rs230915	ACGTTGGATGCAAGAAGCCTTTCAGTTGAG	ACGTTGGATGCCTGACTCTTAGTAAGTCAA	ttttcAGTAAGTCAATAGAGTGCTGC
rs231024	ACGTTGGATGAGCTGGATAAACAGGTCTGG	ACGTTGGATGCCATGCCTGGCCATCTTATT	gGGCCATCTTATTATTATTTAATGA

UEP: Unextended mini-sequencing primer.

**Table 3:** Allele frequencies in cases and controls and odds ratio estimates for TB

SNP ID	Gene	Position	Alleles A^a^/B	*p* value	MAF		HWE *p*	ORs(95%CI)	*p* value
					case	control			
rs6436917	*SP110*	231038160	G/A	0.0221*	0.514	0.445	1.0000	1.32 (1.04,1.67)	0.0221*
rs4327230	*SP110*	231046783	G/C	0.0001*	0.078	0.188	0.7379	0.37 (0.25,0.55)	0.0001*
rs2114591	*SP110*	231050569	C/T	0.0002 *	0.203	0.302	0.3317	0.59 (0.45,0.78)	0.0002 *
rs7580912	*SP110*	231081405	C/T	0.7104	0.399	0.388	0.5182	1.05 (0.82,0.14)	0.7104
rs13422	*PMP22*	15134175	A/C	0.0025*	0.447	0.358	0.4358	1.45 (1.14,1.84)	0.0025*
rs230915	*PMP22*	15148256	G/A	0.2531	0.268	0.299	0.8060	0.86 (0.66,1.12)	0.2531
rs231024	*PMP22*	15157972	A/G	0.0700	0.281	0.332	0.4193	0.79 (0.61,1.02)	0.0700

MAF, minor allelic frequency; HWE, Hardy-Weinberg Equilibrium; ORs, odds ratios; CI: confidence interval.

a Minor allele; *p≤0.05.

Bonferroni's multiple adjustment was applied, with *p*<0.00178 (0.05/28).

We then analyzed associations between *SP110* and *PMP22* genotypes and TB susceptibility (Table 4). Genotypes “GG” in rs6436917 (*p*=0.021, OR: 1.72, 95%CI: 1.08–2.74) and “AA” in rs13422 (*p*=0.002, OR: 2.20, 95%CI: 1.32–3.66) were associated with TB risk. A protective effect was also associated with genotypes “GG” in rs4327230 (*p*=0.035, OR: 0.23, 95%CI: 0.05–1.02) and “CC” in rs2114591 (*p*=0.004, OR: 0.35, 95%CI: 0.17–0.73). The “GG” genotype of rs4327230 remained significant after Bonferroni correction.

**Table 4:** Association between *SP110* and *PMP22* tSNP genotypes and TB risk

SNP_ID	Genotype	No. (Frequency)		OR	95%CI	*p* value
		Case	Control			
rs6436917	GG	61 (28.24%)	76 (19.84%)	1.72	1.08,2.74	0.021
	GA	100 (46.30%)	189 (49.35%)	1.14	0.76,1.70	0.536
	AA	55 (25.46%)	118 (30.81%)	1		-
rs4327230	GG	2 (0.92%)	12 (3.13%)	0.23	0.05,1.02	0.035
	GC	30 (13.82%)	120 (31.33%)	0.34	0.21,0.53	<0.001*
	CC	185 (85.25%)	251 (65.54%)	1		-
rs2114591	CC	10 (4.61%)	39 (10.18%)	0.35	0.17,0.730	0.004*
	CT	68 (31.34%)	153 (39.95%)	0.61	0.43,0.88	0.007*
	TT	139 (64.06%)	191 (49.87%)	1		-
rs7580912	CC	34 (15.67%)	54 (14.10%)	1.13	0.68,1.89	0.639
	CT	105 (48.39%)	189 (49.35%)	1.00	0.69,1.44	0.988
	TT	78 (35.94%)	140 (36.55%)	1		-
rs13422	AA	42 (19.35%)	45 (11.81%)	2.20	1.32,3.66	0.002*
	AC	110 (50.69%)	183 (48.03%)	1.41	0.97,2.06	0.069
	CC	65 (29.95%)	153 (40.16%)	1		-
rs230915	GG	13 (6.10%)	35 (9.26%)	0.62	0.32,1.22	0.165
	GA	88 (41.31%)	156 (41.27%)	0.94	0.66,1.34	0.738
	AA	112 (52.58%)	187 (49.47%)	1		-
rs231024	AA	16 (7.37%)	38 (9.92%)	0.63	0.34.191	0.154
	AG	90 (41.47%)	178 (46.48%)	0.76	0.54,1.08	0.124
	GG	111 (51.15%)	167 (43.60%)	1		-

OR: odd ratio; CI: confidence interval.

*p≤0.05.

Bonferroni's multiple adjustment was applied, with *p*<0.00178 (0.05/28).

We assumed that the minor allele of each SNP was a TB risk factor compared to the wild type allele and analyzed associations between SNPs and TB in various inheritance models (Table 5). Rs6436917 was associated with TB risk in a recessive model (*p*=0.019, OR: 1.59, 95%CI: 1.06–2.39), while rs4327230 was significant in a dominant model (*p*<0.001, OR: 0.33, 95%CI: 0.21–0.51). Two other SNPs were associated with TB in both dominant (rs2114591, *p*<0.001, OR: 0.56, 95%CI: 0.39–0.80; rs13422, *p*=0.013, OR: 1.57, 95%CI: 1.09–2.28) and recessive models (rs2114591, *p*=0.017, OR: 0.43, 95%CI: 0.19–0.89; rs13422, *p*=0.012, OR: 1.79, 95%CI: 1.10–2.91). Rs4327230 and rs2114591 remained significant in the dominant model after Bonferroni correction (*p*<0.00178).

**Table 5:** Association between *SP110* and *PMP22* tSNPs and TB risk based on different inheritance models

SNP_ID	Model	Genotype	Case	Control	OR	95%CI	*p* value
rs6436917	Dominant	G-G/G-A	161	265	1.30	0.88,1.94	0.1656
		A-A	55	118			
	Recessive	G-G	61	76	1.59	1.06,2.39	0.0188*
		G-A/A-A	155	307			
rs4327230	Dominant	G-G/G-C	32	132	0.33	0.27,0.51	0.0001*
		C-C	185	251			
	Recessive	G-G	2	12	0.29	0.03,1.31	0.0847
		G-C/C-C	215	371			
rs2114591	Dominant	C-C/C-T	78	192	0.56	0.393,0.80	0.0008*
		T-T	139	191			
	Recessive	C-C	10	39	0.43	0.19,0.89	0.0166*
		C-T/T-T	207	344			
rs7580912	Dominant	C-C/C-T	139	243	1.03	0.72,1.48	0.8816
		T-T	78	140			
	Recessive	C-C	34	54	1.13	0.69,1.84	0.6017
		C-T/T-T	183	329			
rs13422	Dominant	A-A/A-C	152	228	1.57	1.08,2.28	0.0127*
		C-C	65	153			
	Recessive	A-A	42	45	1.79	1.10,2.91	0.0119*
		A-C/C-C	175	336			
rs230915	Dominant	G-G/G-C	101	191	0.88	0.62,1.25	0.4676
		C-C	112	187			
	Recessive	G-G	13	35	0.64	0.30,1.27	0.1775
		G-C/C-C	200	343			
rs231024	Dominant	A-A/A-G	106	216	0.74	0.52,1.05	0.0748
		G-G	111	167			
	Recessive	A-A	16	38	0.73	0.37,1.37	0.2946
		A-G/G-G	201	345			

OR: odd ratio; CI: confidence interval.

*p≤0.05.

Bonferroni's multiple adjustment was applied, with *p* 0.00178 (0.05/28).

**Figure 1:**
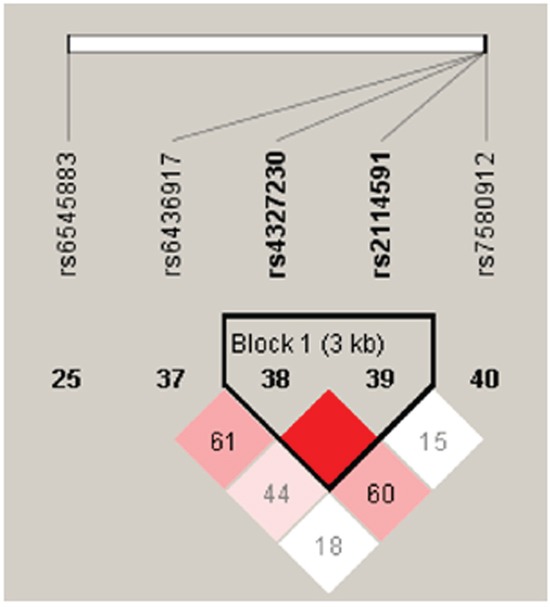
Haplotype block map for *SP110* tSNPs Block 1 includes rs4327230 and rs2114591. The coefficient of linkage disequilibrium (D) between two SNPs is normalized to D' (D/D_max_) (red schemes).

**Figure 2:**
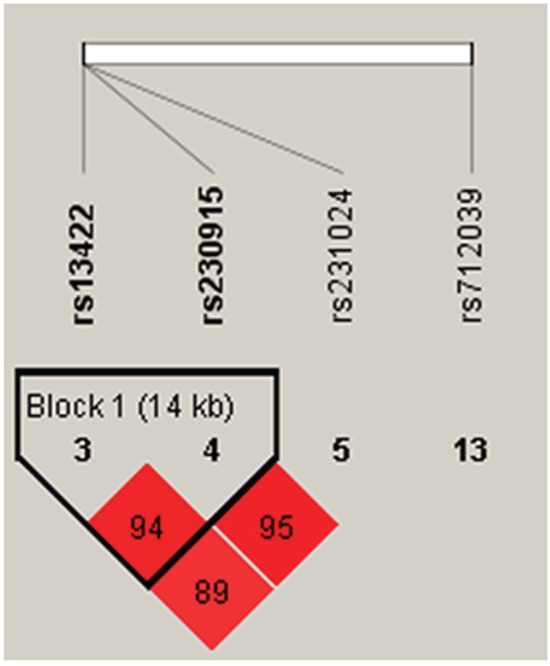
Haplotype block map for *PMP22* tSNPs Block 2 includes rs13422 and rs230915. The coefficient of linkage disequilibrium (D) between two SNPs is normalized to D' (D/D_max_) (red schemes).

We detected two blocks in *SP110* and *PMP22* via haplotype analysis (Figure 1 & 2). Block 1 in *SP110* included rs4327230 and rs2114591 and Block 2 in *PMP22* included rs13422 and rs230915. The global result for Block 1 (rs4327230 and rs2114591) was: total case = 434, total control = 766, global χ^2^ = 26.416 while df = 2, Pearson's *p* value<0.001. The result for Block 2 (rs13422 and rs230915) was: total case = 434, total control = 766, global χ^2^=10.567 while df = 3, Pearson's *p* value=0.014. We did not perform Fisher's exact probabilities because the total sample size exceeded 40 and all theoretical frequencies were >1.

Further analyses of associations between *SP110* and *PMP22* haplotypes and TB risk showed that haplotype “GC” in Block 1 was protective against TB (*p*<0.001, OR: 0.36, 95%CI: 0.24–0.54), which remained significant after Bonferroni correction (*p*<0.00178) (Table 6). Both haplotypes “CA” (*p*=0.024, OR: 0.71, 95%CI: 0.53–0.96) and “CG” (*p*=0.021, OR: 0.70, 95%CI: 0.52–0.95) were also protective against TB. These results suggested that rs13422 allele A might be a risk factor for TB susceptibility.

**Table 6:** Association of haplotype frequencies with TB risk in cases and controls

Block	SNPs	Haplotypes	Frequencies		χ^2^	Pearson's *p*	OR	95%CI
			case	control				
1	rs4327230 | rs2114591	CT	0.797	0.698	-	-	1 (reference)	-
		GC	0.078	0.188	26.365	<0.001*	0.36	0.24-0.54
		CC	0.124	0.114	0.314	0.84	0.96	0.68-1.37
2	rs13422 | rs230915	AA	0.429	0.351	-	-	1 (reference)	-
		CA	0.302	0.350	2.869	0.024*	0.71	0.53-0.96
		CG	0.251	0.291	2.203	0.021*	0.70	0.52-0.95
		AG	0.018	0.008	2.518	0.190	2.60	0.62-10.90

OR: odd ratio; CI: confidence interval

Bonferroni's multiple adjustment was applied, with *p*<0.00178 (0.05/28).

## DISCUSSION

Multiple groups have investigated associations between *SP110* and TB susceptibility in a variety of populations, but these study results are generally considered controversial [8-10, 12, 16-18]. In the current case-control study, we investigated seven total SNPs in a Tibetan population and found that three in *SP110* (rs6436917, rs4327230, rs2114591) and one in *PMP22* (rs13422) were associated with TB risk. To the best of our knowledge, this is the first study to demonstrate an association between *PMP22* and TB, and the first study of *SP110* in Tibetans. We found that the “GC” haplotype in *SP110* was protective against TB, with a 64% reduction in disease risk. “CA” and “CG” in *PMP22* were also associated with a protective effect.

Two *SP110* SNPs, rs2114591 and rs6436917, were particularly notable in this study. The rs2114591 C allele in intron 11 of *SP110* greatly reduced TB risk in the study population. However, a previous study by Abhimanyu, *et al.* [8] showed no association between rs2114591 and TB in lymph node tuberculosis in north Indians. Moreover, neither Thye, *et al.* [12] nor Png, *et al.* [17] found any association between rs2114591 and TB risk in Indonesians or the republic of Ghana, respectively. Rs6436917 was also associated with TB in our study. Abhimanyu, *et al.* [8] did not detect this SNP in their study, but instead associated another SNP 3636 bp upstream of rs6436917, rs6436915, with TB risk (OR=3, 95%CI, 1.3-6.3; *p*=0.0057). Our study and others indicate that gene expression may differ among races, and this should be taken into consideration when determining an individual patient's susceptibility to TB.

*PMP22* encodes an integral membrane protein that is a major component of myelin in the peripheral nervous system. It is expressed in Schwann cells and also in non-neural cells such as the lung epithelium, and influences cell proliferation [19]. In the current study, we identified the rs13422 A allele in the *PMP22* 3′UTR as a TB risk factor in a Tibetan population. This is the first study to associate *PMP22* and TB risk.

TB elicits a kind of type IV allergic reaction (also called delayed type hypersensitivity, DTH). The main pathological processes involved include exudation, alteration and proliferation, which depend on both the host immune system and *M. tuberculosis* strain virulence. Mouse models have been employed to explore the impacts of *Ipr1*, encoded within the *sst1* locus, on TB susceptibility. Kramnik [7] showed that *sst1* gene expression may be regulated by macrophages in a cell-autonomous manner. Chackerian, *et al.* [20] also found increased numbers of IFN-γ-producing CD4^+^ T cells in the lungs of *sst1*-resisitant C57BL/6 inbred mice, and conjectured that TB susceptibility was related to the ability to recruit mycobacteria-specific IFN-γ-producing Th1 cells to the lungs. Mechanisms of TB resistance via *Ipr1* and *sst1* have been extensively explored, but studies of *SP110* with respect to TB in humans are rare, and no work had yet addressed *PMP22*, which is primarily associated with neural diseases, in TB. Though we found that *PMP22* is a risk factor for TB, the mechanisms by which *PMP22* affects immunity are unknown and require further study.

The Bonferroni correction is one of the most important methods used to address false discovery rates resulting from multiple testing. We found that SNPs rs4327230 and rs2114591 remained significant after Bonferroni correction, while rs6436917 and rs13422 were not associated with TB. This may due to our strict SNP filtering criteria and small sample size. Additionally, the Bonferroni correction adjusts the value of alpha based on the number of tests performed, and is thus conservative; in some cases, truly significant differences may be deemed non-significant as a result of type II errors [21].

Our study faced some intrinsic limitations. Our patient sample size (217 cases and 383 controls) was not large enough for the association analysis. Other studies included more than 1000 samples [22], and their SNP-TB association findings are therefore more reliable. Additionally, the case groups we selected included patients diagnosed with clinical tuberculosis (sputum smear-positive), regardless of their typing, which could potentially reduce the validity of our conclusions.

Our study provides new evidence supporting the importance of host genetic variability in TB susceptibility. We identified *SP110* genotypes that appear protective against TB, and identified *PMP22* genotypes as risk factors in TB susceptibility. Our study may help to identify those TB-affected individuals most susceptible to disease, and to improve patient-specific clinical TB diagnosis.

## MATERIALS AND METHODS

### Ethics statement

We strictly obeyed the World Medical Association Declaration of Helsinki when using human tissue and signing the study protocol with subjects, which was approved by the Ethical Committee of Xi'an Jiaotong University. Each participant provided written, informed consent.

### Subjects

All participants in our study were Tibetan Chinese. A total of 143 TB patients were consecutively recruited between March 2013 and June 2015 in the Tangdu Hospital, affiliated with The Fourth Military Medical University in Xi'an city, China. Patients were diagnosed with TB using symptoms and signs, chest X-ray, sputum smear test for acid fast bacilli (AFB) and *M. tuberculosis* sputum culture. All patients were HIV negative, which was confirmed by the HIV serology test. There were no age, sex or TB classification restrictions when enrolling the case group. Controls were healthy people receiving physical examinations in other clinical departments of Tangdu Hospital. Healthy controls did not have respiratory disease, immune system disease or any other infectious disease, such as acquired immunodeficiency syndrome (AIDS), that could have affected our study results. Peripheral blood was collected from both cases and controls for DNA extraction.

### Clinical data and patient demographics

A standardized epidemiological questionnaire was provided to all subjects to collect basic demographic information, including sex, age, residence, educational status, history of family cancer, history of smoking, alcohol consumption and TB contact history. Plasma carcinoembryonic antigen and alpha-fetoprotein levels were determined to ensure that no controls suffered any cancer.

### SNP selection and genotyping

Candidate SNPs in the *SP110* and *PMP22* genes were selected from previous publications that associated polymorphisms with TB [23, 24]. SNPs with minor allele frequencies (MAF) > 5% in the HapMap CHB population were selected. We validated four SNPs in *SP110* and three in *PMP22*. The GoldMag-Mini Purification Kit (GoldMag Co. Ltd. Xian city, China) was used to extract genomic DNA from whole blood samples. DNA concentration was measured using a DU530 UV/VIS spectrophotometer (Beckman Instruments, Fullerton, CA, USA). Using MassARRAY Assay Design 3.0 software (Sequenom, San Diego, CA, USA), we designed a multiplexed SNP MassEXTENDED assay. SNPs were genotyped using the standard protocol recommended by the MassARRAY RS1000 (Sequenom) manufacturer and data were analyzed using Typer 4.0 Software (Sequenom).

### Statistical analysis

We used Microsoft Excel and SPSS 16.0 (SPSS, Chicago, IL) statistical packages to perform statistical analyses. All *p*-values were two-sided and *p*<0.05 was considered statistically significant. A *t* test and Chi-square test were performed to compare sex and age differences between cases and controls. Fisher's exact test was applied to each SNP in the controls to test for departure from Hardy–Weinberg Equilibrium (HWE). Odds ratios (ORs) and 95% confidence intervals (CIs) for the allele and genotype frequencies were calculated using Pearson Chi-square test adjusted by age and gender. Cochran-Armitage trend test was performed to determine associations between SNPs and TB. Sex and age subgroups were not separated due to sample size limitations.

PLINK software (http://pngu.mgh.harvard.edu/purcell/plink/) was used to assess SNP associations with TB risk in different genetic models (dominant and recessive). We used unconditional logistic regression analysis to calculate ORs and 95% CI adjusted for age and gender. Pairwise linkage disequilibrium and haplotype constructions were performed using HAPLOVIEW 4.1 (http://broad.mit.edu/mpg/haploview) [25]. All *p* values were Bonferroni corrected, and statistical significance was set at *p*<0.00178 (0.05/28).
